# Quantitative analysis of dense-core vesicle fusion in rodent CNS neurons

**DOI:** 10.1016/j.xpro.2021.100325

**Published:** 2021-02-03

**Authors:** Alessandro Moro, Rein I. Hoogstraaten, Claudia M. Persoon, Matthijs Verhage, Ruud F. Toonen

**Affiliations:** 1 Department of Functional Genomics, Center for Neurogenomics and Cognitive Research (CNCR), Vrije Universiteit (VU) Amsterdam, de Boelelaan 1085, 1081 HV Amsterdam, the Netherlands; 2 Department of Clinical Genetics, UMC Amsterdam, the Netherlands

**Keywords:** Cell culture, Cell membrane, Microscopy, Neuroscience, Molecular/chemical probes

## Abstract

Neuropeptides are essential signaling molecules secreted by dense-core vesicles (DCVs). They contribute to information processing in the brain, controlling a variety of physiological conditions. Defective neuropeptide signaling is implicated in several psychiatric disorders. Here, we provide a protocol for the quantitative analysis of DCV fusion events in rodent neurons using pH-sensitive DCV fusion probes and custom-written analysis algorithms. This method can be used to study DCV fusion mechanisms and is easily adapted to investigate fusion principles of other secretory organelles.

For complete details on the use and execution of this protocol, please refer to Persoon et al. (2019).

## Highlights

• Protocol for quantitative analysis of neuropeptide exocytosis in rodent CNS neurons
• Generation and lentiviral infection of neurons on prepatterned astrocyte microdot islands
• Visualization of neuronal DCV fusion events using pH-sensitive fusion probes
• Quantitative analysis of fusion events using custom-written algorithms

## Graphical Abstract

**Figure:**
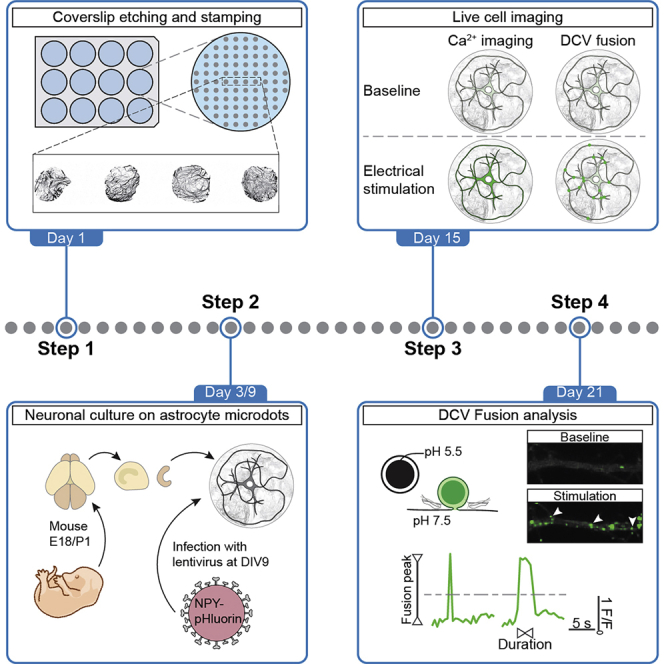

## Before you begin

This protocol describes the experimental steps to quantify DCV fusion events in single cultured neurons grown on microdot islands of astrocytes using pH-sensitive DCV fusion probes. The protocol covers the production of lentiviral particles, preparation of single neuron cultures on astrocyte microdots, image acquisition, and data analysis using custom-written algorithms.

### Lentivirus particle production

**Timing: 3 days**

For efficient infection of cultured neurons use lentiviral infection to express your gene of interest. In this protocol we use the DCV fusion probes NPY-pHluorin and BDNF-pHluorin, and synapsin-mCherry as live synapse marker. All constructs are driven by a human synapsin promoter for neuron specific expression.

These steps describe lentiviral particle production according to Naldini et al. (1996). We use a 2^nd^ generation lenti viral production system (see https://www.addgene.org/guides/lentivirus/ for info on the use of 2^nd^ or 3^rd^ generation systems).

1. Plate 293T cells from an 80% confluent T175 flask in 245 mm square tissue culture (TC) treated-dishes in DMEM complete (please refer to “Materials and equipment” for media recipes). Incubate in a 37°C, 5% CO_2_ incubator for 1 day, or until 50% confluence.

**CRITICAL:** 293T cell passage number should be < 25.

2. For each lenti viral batch (lenti-hSyn-NPY-pHluorin, lenti-hSyn-BDNF-pHluorin, or lenti-hSyn-synapsin-mCherry), transfect cells with a plasmid mix in PBS containing a VSV-G envelope plasmid (e.g., p.MD2.G, 11.4 μg), packaging plasmid (e.g., pCMVΔR8.2, 22.4 μg) and a transfer plasmid containing your gene of interest (e.g., pRRL, 45.8 μg) using polyethyleneimine (PEI; 240 μg). Make up a total volume of 5 mL PBS per plate with a DNA to PEI ratio of 1:3.
3. Replace media with 100 mL Opti-MEM without fetal calf serum (FCS) after 1 day.
4. Harvest conditioned medium 40 h after transfection.
5. Centrifuge supernatant using an Amicon spin filter (100 kDa cutoff) at 4,000 × *g* for 20–30 min.
6. Transfer the ± 150 μL of concentrated virus to a tube, dilute in 1 mL PBS and filter sterilize through a 0.2 μm syringe filter.
7. Typical viral titers obtained with this method range from 5 × 10^8^ IFU/mL to 1 × 10^9^ IFU/mL.

**CRITICAL:** Viral particles should be aliquoted in single use 0.5 mL tubes, 25–50 μL per tube, and stored at −80°C. Do not re-freeze. Thawed aliquots can be stored at 4°C for up to 4 weeks without loss of efficiency.

### Coverslip preparation for neuronal culture on astrocyte microdots

**Timing: 2 days**

8. Etch 18 mm glass coverslips in 1 N HCl for 12–16 h, wash with H_2_O followed by 1 h in 1 N NaOH.
9. Rinse extensively with H_2_O and briefly in 70% EtOH. Place coverslips, once air dried, in 12-well plates.
10. Apply 0.5 mL of agarose type II-A (1.5 gr/l in H_2_O) to cover the entire surface of each coverslip. Remove agarose with a 1 mL pipet within 30 s and let the coverslip air dry.
11. Prepare the Poly-D-lysine (PDL)-collagen-acetic acid-mix (1:1:3). PDL (0.5 mg/mL; stored at −20°C), collagen (∼3.5 mg/mL; stored at 4°C), acetic acid (17 mM; stored at 4°C).
12. Put 500 μL of mix onto a filter paper (3 mm cellulose chromatography paper, Whatman) and suck off the extra fluid, the paper should be wet, but not soaked.
13. Put a rubber stamp containing a grid of 250 μm diameter dots (Burgalossi et al., 2012) on the paper (do not push) and then on the agarose-treated coverslip (give a little pressure).
14. UV-sterilize the plates for 30 min. Plates can be stored up to 2 weeks at 19°C–22°C in a sterile environment.

### Culture single primary hippocampal neurons on astrocyte microdots

**Timing: 3 weeks**

Astrocyte microdot and primary hippocampal culture preparations have been extensively described (Burgalossi et al., 2012). This is a brief overview highlighting the most important steps.

**Figure 1:**
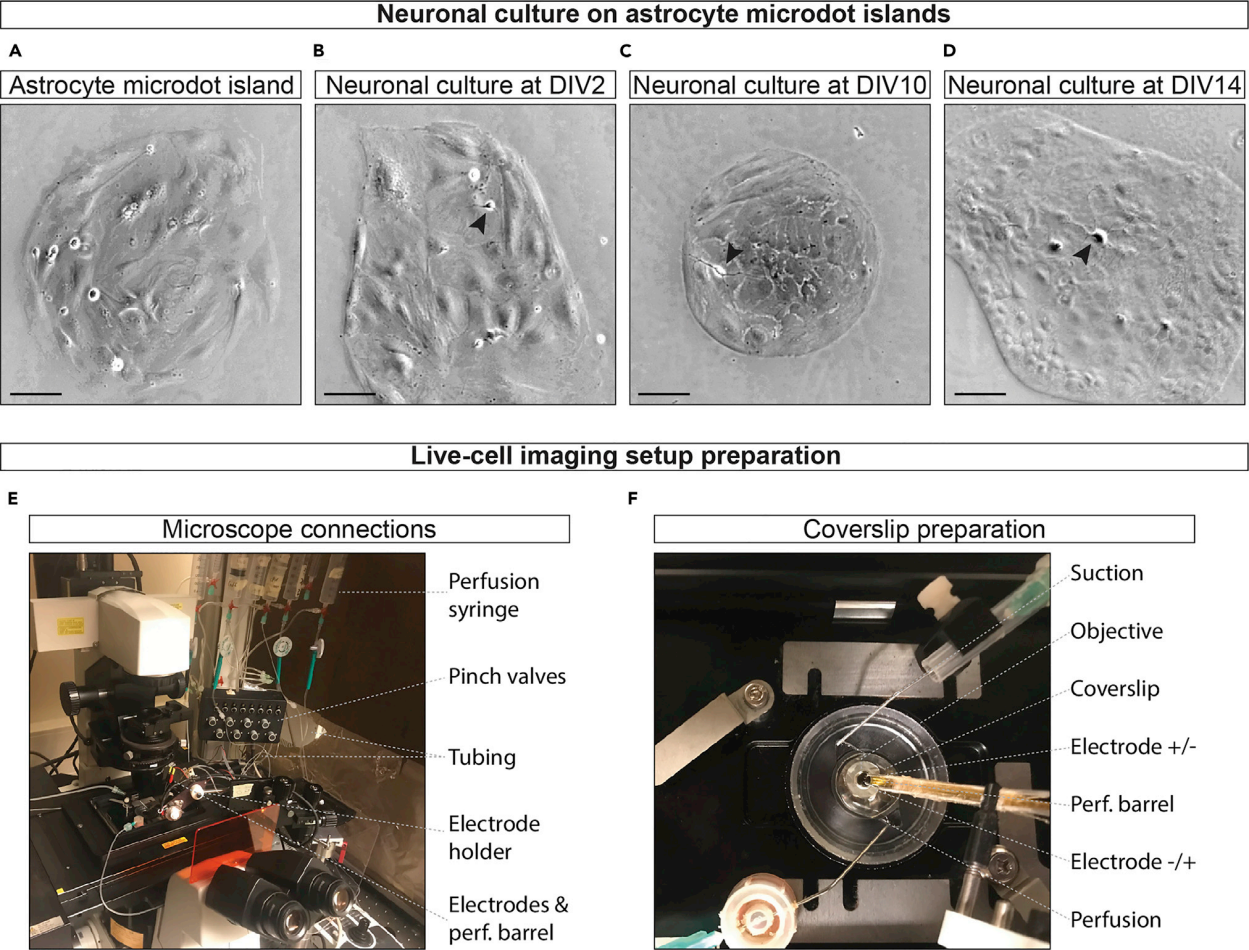
Neuronal culture and microscope setup (A) Example of astrocyte microdot island. Scale bar, 100 μm. (B) Example of hippocampal neuron, indicated by the arrowhead, 2 days after plating (DIV2) on astrocyte microdot. Scale bar, 100 μm. (C) Example of hippocampal neuron (arrowhead) at DIV10 on astrocyte microdot. Scale bar, 100 μm (D) Example of hippocampal neuron (arrowhead) at DIV14 on astrocyte microdot. Scale bar, 100 μm. (E) Live-cell imaging setup with pinch-valve controlled perfusion system, electrodes, and perfusion barrel. (F) Example of a coverslip mounted on the microscope stage with perfusion and suction needles, electrodes, and barrel system to deliver Tyrode’s NH_4_Cl, or other solutions.

***Note:*** DMEM+ should be replaced with NB+ medium on the day of neuronal culture, or 1 day before.

15. Astrocyte cell preparation: Under a dissection microscope, isolate cortices of two P1 rat pups in Hanks+ medium.
16. Incubate for 45 min in 5 mL of papain containing ES medium at 37°C with gentle agitation.
17. Remove ES and add 5 mL of IS medium and incubate for another 15 min. Remove IS and add 2 mL DMEM+ medium. Triturate cortices with a fire-polished Pasteur pipette.
18. Plate the single cell suspension in T175 flasks in 35 mL DMEM+ and incubate at 37°C, 5% CO_2_.
19. Refresh medium after 1 day.
20. When astrocytes are confluent (± 7 days), dissociate the cells from the flask with trypsin-EDTA, count the cells and plate at a density of 6,000 cells/well of a 12 well plate in DMEM+.
21. Incubate the astrocyte culture in a 37°C, 5% CO_2_ incubator for 3–4 days, or until confluent on the microdots (Figure 1A). Replace DMEM+ with NB+ medium.

22. Under a dissection microscope, isolate E18-P1 mouse hippocampi in Hanks+ medium. Add 0.025% Trypsin to the Hanks+ solution and incubate hippocampi for 20 min at 37°C, triturate the hippocampi, count the cells and plate neurons at a density of 1,000 cells/well of a 12 well plate in NB+ medium.
23. Incubate astrocyte-neuron co-cultures at 37°C, 5% CO_2_ for up to 21 days.

### Infect neurons with DCV fusion reporter constructs

**Timing: 5 min**

This step labels neuronal DCVs with super-ecliptic pHluorin to monitor DCV fusion.

24. At day-in-vitro (DIV) 9–10, check the quality of island cultures under a bright-field microscope. The majority of the astrocyte islands should be intact and confluent and contain no more than one hippocampal neuron (Figure 1C).
25. Infect neurons by adding 0.5–3 μL of lentivirus particles encoding for BDNF- or NPY-pHluorin to label DCVs and synapsin-mCherry to label synapses into each well.

**CRITICAL:** Titrate new lentiviral batches by infecting DIV9–10 neurons with a limiting dilution series. In our hands, 0.5 to 3 μL of a virus solution (∼5 × 10^8^ IFU/mL) prepared according to our lentivirus particle production protocol will result in infection of >90% of neurons imaged using a fluorescence microscope at DIV14. For each lentiviral batch the optimal concentration needs to be assessed. Batches will remain stable for at least 12 months at −80°C and for up to 4 weeks when stored at 4°C. For lenti-hSyn-NPY-pHluorin and lenti-hSyn-BDNF-pHluorin, many moving and stationary puncta should be visible upon Tyrode's NH_4_Cl perfusion. For lenti-hSyn-synapsin-mCherry, immobile puncta (presynaptic terminals) should be visible. Select the optimal viral titer for use in live imaging experiments.

26. Leave 1 or 2 coverslips per condition uninfected. These will be used for Fluo5 Ca^2+^ imaging at DIV14.

***Optional:*** Infection of additional lentiviral constructs (Cre, rescue constructs) can be performed at earlier or later timepoints. However, the total volume of viral solution should not exceed 20 μL.

### Prepare solutions for live-cell imaging

**Timing: 30 min**

27. Prepare Tyrode's solution and Tyrode's solution containing NH_4_Cl (refer to “Materials and equipment” for recipes).
  a. Dissolve all salts in dH_2_O at 19°C–22°C.
  b. Adjust the pH to 7.4 with 1 N NaOH.
  c. Check the osmolarity and adjust to 280 mOsmol with glucose.
  d. Filter sterilize and store at 4°C.
  e. Bring to 19°C–22°C before live-cell imaging.
28. Prepare 2 mM Fluo5-AM aliquots of 5 μL by dissolving Fluo5-AM in DMSO.

## Key resources table

**Table:** 

REAGENT or RESOURCE	SOURCE	IDENTIFIER
**Chemicals, peptides, and recombinant proteins**		

Agarose type II-A	Sigma	Cat# A9918
Laminin	Sigma	Cat# L2020
Poly-D-lysine	Sigma	Cat# P6407
Rat tail collagen	BD Biosciences	Cat# 354236
Acetic acid	VWR	Cat# 20104.334
B-27	Gibco	Cat# 17504-044
PBS	Gibco	Cat# 14190-094
Polyethyleneimine	Polysciences	Cat# 23966-1
Opti-MEM	Fisher	Cat# 12087549
Hanks	Sigma	Cat# H9394
HEPES 1 M	Gibco	Cat# 15630-056
DMEM + UltraGlutamine I	Lonza	Cat# LO BE12-604F/U1/12
FCS	Gibco	Cat# 10270
NEAA	Life Technologies	Cat# 11140-035
Penicillin/streptomycin	Gibco	Cat# 15630-056
L-Cysteine	Sigma	Cat# C7352
EDTA	AppliChem	Cat# A2937 0500
Neurobasal medium	Gibco	Cat# 21103-049
Glutamax	Gibco	Cat# 35050-038
Papain	Worthington	Cat# 3127
Trypsin-EDTA	Gibco	Cat# 25300-054
Trypsin	Gibco	Cat# 1509-046
Albumin	AppliChem	Cat# A1391 0100
Trypsin inhibitor	Sigma	Cat# T9523
NaOH	Sigma	Cat# S5881
HCl	Sigma	Cat# 38283
EtOH	VWR	Cat# 83804.360
NaCl	Fisher	Cat# 15673410
KCl	SERVA	Cat# 7447-40-7
CaCl_2_·2H_2_O	Sigma	Cat# C7902
MgCl_2_·6H_2_O	Sigma	Cat# M9272
HEPES (1 M)	Fisher	Cat# 15630-056
Glucose-H_2_O	VWR	Cat# 1040741000
NH_4_Cl	Sigma	Cat# 12125-02-9
Fluo-5F-AM	Molecular Probes	Cat# F14222
Dimethyl sulfoxide (DMSO)	Sigma	Cat# D2438

**Experimental models: cell lines**		

Mouse: primary neurons from hippocampus, cortex, or striatum	N/A	N/A
Rat: primary astrocytes	N/A	N/A
293T cells	ATCC	CRL-11268

**Experimental models: organisms/strains**		

Mouse: *Rab3ABCD* null embryonic day 18 (E18) pups	Schlüter et al., 2004	N/A
Mouse: *Rim1/2* conditional null mutant E18 pups	Kaeser et al., 2011; Kaeser et al., 2008	N/A
Rat: Wistar (Crl:WI) P1 pups	Charles River	Strain code: 003

**Recombinant DNA**		

hSyn(pr):NPY-pHluorin	Van de Bospoort et al., 2012	N/A
hSyn(pr):rBDNF-pHluorin	de Wit et al., 2009	N/A
hSyn(pr):Synapsin-mCherry	(Persoon et al., 2019)	N/A
p.MD2.G (encoding VSV-G envelope)	Naldini et al., 1996	Addgene: #12259
pCMVΔR8.2 (2^nd^ gen. packaging encoding HIV-1 Gag, Pol, Tat, and Rev proteins)	Naldini et al., 1996	Addgene: #12263
pRRL (2^nd^/3^rd^ gen. transfer)	Naldini et al., 1996	Addgene: #12252

**Software and algorithms**		

MATLAB	MathWorks	https://mathworks.com
ImageJ	NIH	https://imagej.net/ RRID:SCR_003070
DCV_pHluorin	N/A	https://github.com/alemoro/
Microsoft Excel	Microsoft	https://www.microsoft.com
NIS Elements	Nikon Instruments	https://www.nikoninstruments.com/Products/Software RRID:SCR_014329

**Other**		

0.2 μm syringe filters	Fisher	Cat# 10331075
T175 flask	Greiner	Cat# 690175
TC-treated culture dishes	Fisher	Cat# 10350602
Amicon spin filter	Millipore	Cat# UFC910096
3 mm cellulose chromatography paper, Whatman	VWR	Cat# 514-8013
18 mm glass coverslip	VWR	Cat# 631-1342
12 well plates	Greiner	Cat# 665165
NikonTi-E Eclipse	Nikon	https://nikon.com
EMCCD DU-897 camera	Andor	https://andor.com
Master-8	AMPI	https://ampi.co.il
Stimulus isolator A-395	WPI	https://wpiinc.com
Pinch valve	N/A	N/A
50 mL perfusion syringes	VWR	Cat# 613-5398
Exadrop	B. Braun	Cat# 4061306
Tubing	Sigma	Cat# 58703
Luer Lock male	Brunschwig	Cat# CT58.1
Luer Lock female	Brunschwig	Cat# CT62.1
Glass capillary	GmbH	Cat# GB150F-8P
Platinum electrodes	N/A	N/A
Dissection microscope	Nikon	SMZ745

**Deposited data**		

DCV_pHluorin code	Persoon et al., 2019	https://github.com/alemoro

## Materials and equipment

**Table:** DMEM complete medium (DMEM+)

Reagent	Final concentration (mM or μM)	Volume (mL)
DMEM + UltraGlutamine I	1×	440
FCS	10%	50
NEAA	1%	5
Penicillin/streptomycin	50,000 U/mL	5
**Total**	**n/a**	**500**

Store at 4°C for up to 1 month. Prepare 50 mL aliquots to reduce rounds of heating (37°C) and cooling, and risk of contamination.

**Table:** Hanks+

Reagent	Final concentration (mM or μM)	Volume (mL)
Hanks	1×	990
HEPES 1 M	10 mM	10
**Total**	**n/a**	**1,000**

Store at 19°C–22°C in the dark for up to 24 months.

**Table:** Enzyme solution (ES)

Reagent	Final concentration	Volume (mL)
DMEM + UltraGlutamine I	1×	245
L-Cysteine	50 mg	n/a
CaCl_2_	100 mM	2.5
EDTA	50 mM	2.5
**Total**	**n/a**	**250**

Store at −20°C in 5 mL aliquots for up to 12 months.

Preparation:

- Defrost ES (5 mL) and add 20–25 U/mL papain.
- Incubate at 37°C until papain is dissolved (solution should be clear).
- Bubble 15 min with carbogen (solution should go from purple to more pinkish) and sterilize with 0.2 μm filter before use.

**Table:** Inhibitor solution (IS)

Reagent	Final concentration	Volume (mL)
DMEM + UltraGlutamine 1	1×	225
FCS	10%	25
Albumin	625 mg	n/a
Trypsin inhibitor	625 mg	n/a
**Total**	**n/a**	**250**

Store at −20°C for up to 12 months.

**Table:** Complete neurobasal medium (NB+)

Reagent	Final concentration	Volume (mL)
Neurobasal medium	1×	479.25
B-27 Supplement	2%	10
HEPES 1 M	17 mM	9
Glutamax	1%	1.25
Penicillin/streptomycin	50,000 U/mL	0.5
**Total**	**n/a**	**500**

Store at 4°C for up to 1 month. Prepare 50 mL aliquots to reduce rounds of heating (37°C) and cooling, and risk of contamination.

**Table:** Tyrode’s imaging medium

Reagent	Final concentration (mM)	Volume (mL)
NaCl	119	n/a
KCl	2.5	n/a
CaCl_2_	2	n/a
MgCl_2_	2	n/a
HEPES	25	n/a
Glucose	30	n/a
**Total**	**n/a**	**n/a**

Store at 4°C for up to 6 months.

**Table:** Tyrode’s NH_4_Cl medium

Reagent	Final concentration (mM)	Volume (mL)
NaCl	69	n/a
KCl	2.5	n/a
CaCl_2_	2	n/a
MgCl_2_	2	n/a
NH_4_Cl	50	n/a
HEPES	25	n/a
Glucose	30	n/a
**Total**	**n/a**	**n/a**

Store at 4°C for up to 6 months.

## Step-by-step method details

### Microscope preparation

**Timing: 10 min**

Here, directions are provided to setup a Nikon Ti-E Eclipse inverted microscope system for live-cell imaging experiments. These steps are similar for other microscopes and acquisition software such as Metamorph, AxioVision, and Olympus Zen. Please refer to the manufacturer's instructions how to send a TTL signal to the Master-8.

Live imaging experiments are performed on a Nikon Ti-E Eclipse inverted microscope system fitted with a Confocal A1R (LU4A Laser) unit and an EMCCD (Andor DU-897), a 40× oil objective (NA 1.3) and appropriate filter sets. NIS Elements software (version 4.30) controls the microscope and image acquisition. Electrical field stimulation is applied via two parallel platinum electrodes controlled by a Master-8 (AMPI) connected to a stimulus isolator (A-395, World Precision Instruments). Superfusion of NH_4_Cl containing Tyrode’s through a glass capillary placed just above the neuron is controlled with a pinch valve (Figure 1E).

1. In the image acquisition software NIS Elements open ND Acquisition and select time. Set the interval as time between frames to 0.5 s for 2 Hz image acquisition, and the duration of the image acquisition.
2. Set TTL out to trigger the Master-8 at the start of the image acquisition.
3. Set up a Master-8 paradigm (Figure 2):
  a. Start with a delay of 30 s to record baseline fluorescence.
  b. Send 50, 1 ms pulses at 50 Hz to the stimulus isolator for 16 times with 0.5 s between bursts.
  c. Add a delay of 26 s to record post-stimulus fusion events.
  d. Send a 10 s pulse to the pinch valve controller to start superfusion of NH_4_Cl containing Tyrode’s 26 s after stimulation.

**Figure 2:**
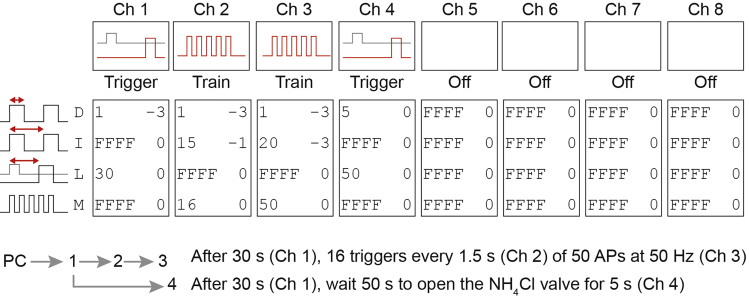
Master 8 setup Graphical representation of the Master 8 interface and stimulation paradigm used in this protocol. Channel 1 is connected to the computer (PC), channel 3 is connected to the stimulus isolator, and channel 4 is connected to the pinch valve system for Tyrode’s NH_4_Cl application. Channels 2, 5, 6, 7, 8 are not connected to an external device. Channel 1 receives the TTL signal from the PC at the start of the time-lapse recording. After a 30 s (L) delay, channel 1 delivers a 1 ms (D) trigger to channel 2 and channel 4. Channel 2 responds with 16 (M) 1 ms (D) pulses every 1.5 s (I) that triggers channel 3 with 50 (M) 1 ms (D) pulses every 20 ms (I). Thus, the communication between channel 2 and 3 generates the 16 trains of 50 action potentials at 50 Hz. Channel 4 responds 50 s (L) after input from channel 1 by delivering a 5 s (D) trigger to the pinch valve to start Tyrode's NH_4_Cl perfusion.
4. Fill Tyrode’s and Tyrode’s NH_4_Cl syringes for gravity flow delivery via software controlled pinch valves (Figure 1E).

### Live-cell imaging, Ca^2+^ imaging

**Timing: 30 min to 1 h**

This step visualizes intracellular Ca^2+^ elevations upon stimulation and subsequent buffering from the cytosol. It provides a read out of the culture’s physiological state and is typically performed at the start of each DCV fusion imaging session.

5. Incubate one coverslip containing uninfected DIV14 neurons with Fluo5-AM (2 μΜ final concentration) for 10 min at 37°C, 5% CO_2_.
6. Place the coverslip into an imaging chamber and add 500 μL of Tyrode’s solution.

7. Prepare neurons for live-cell imaging at the microscope setup (Figure 1F).
  a. Place the imaging chamber in the stage holder and bring the neurons in focus using a 40× oil objective in DIC or fluorescence mode (Figure 1D).
  b. Establish Tyrode's perfusion of 0.5 mL/min by placing one perfusion needle at one end and a suction needle at the opposite end of the imaging chamber.
  c. Place two platinum electrodes connected to the stimulus isolator at opposite sides of an astrocyte microdot containing a single neuron.
    **CRITICAL:** Make sure that the distance between the electrodes spans one astrocyte microdot (± 0.5mm) and are placed in such a way that the center of your field of view lies between the electrodes. Lower the electrodes as far as possible, just above the glass coverslip but do not touch the coverslip.
  d. Adjust the 488nm laser power and camera exposure time for optimal dynamic range.
    **CRITICAL:** Make sure that the camera pixels are far from saturation since stimulation will typically induce a 100-fold increase in fluorescence.

8. Start time-lapse recording (image acquisition at 2Hz, or higher) and trigger the Master-8 via TTL pulse.
9. Repeat steps 7c–8 for a maximum of 5 times, or for no longer than 1 h.

**CRITICAL:** Make sure to leave at least one astrocyte microdot between a previously stimulated neuron and the next to guarantee that each neuron is naive at the start of each recording.

**Figure 3:**
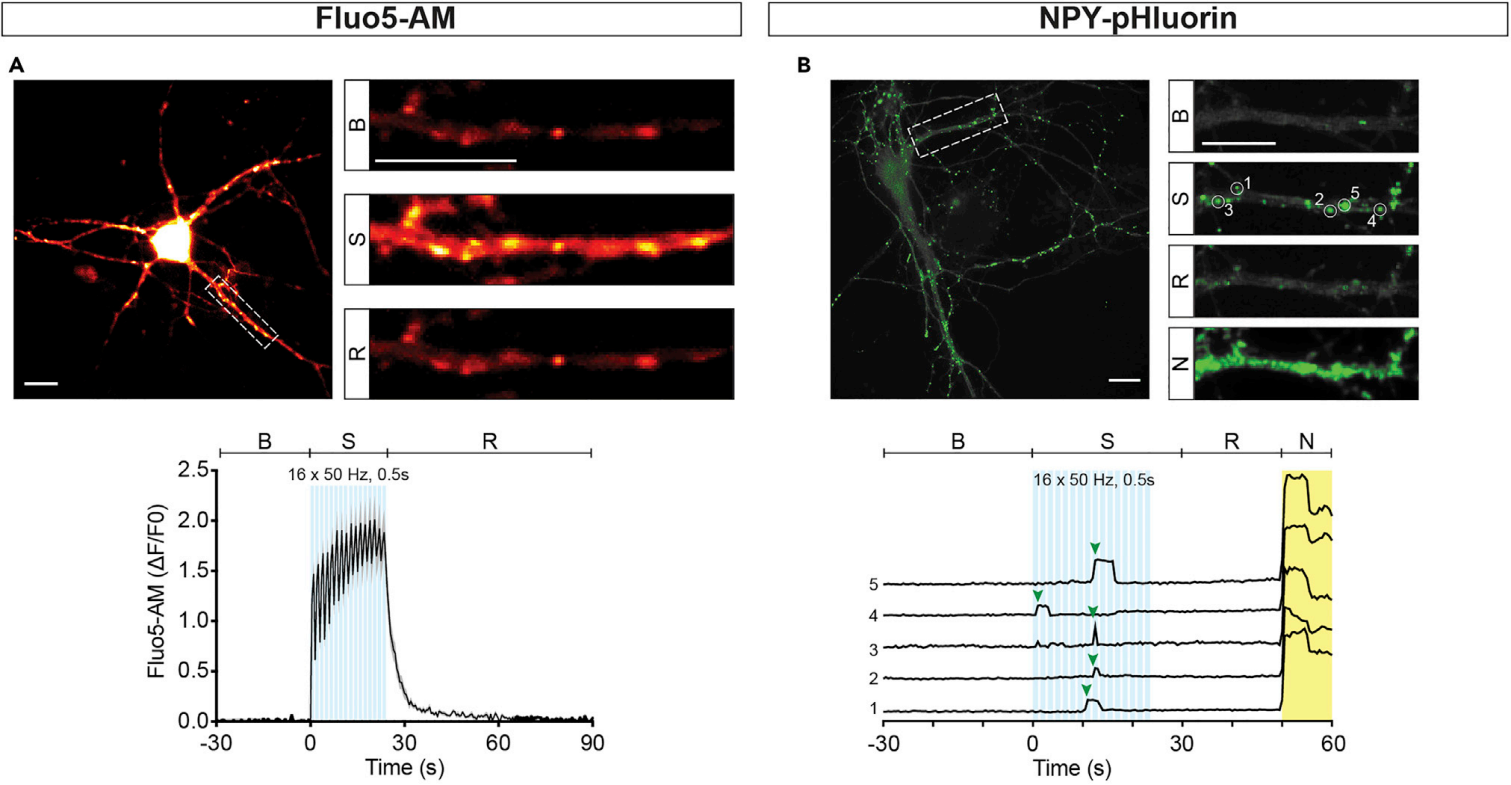
Neuronal live-cell imaging (A) Top, typical neuron after incubation with the Ca^2+^ reporter Fluo5-AM. Insets show zoom of a neurite before field stimulation (B), during stimulation (S) and 2 s after stimulation (R). Bottom, average Fluo5-AM signal during a live-cell imaging experiment consisting of 30 s baseline recording (B), 16 trains of 50 AP at 50 Hz stimulation (S, light blue bars), and 50 s recovery (R). Note the sharp rise and fall of the calcium signal. Scale bar, 20 μm in all. (B) Top, typical neuron with NPY-pHluorin fusion events, calculated using the Standard Deviation Z-projection option in Fiji, with a zoom of a neurite before field stimulation (B), during stimulation (S), after stimulation (R), and during Tyrode’s NH_4_Cl superfusion (N). Bottom, five example intensity traces showing typical NPY-pHluorin fusion events (start indicated with green arrow head). The intensity was calculated in 3 × 3 pixel ROIs. Scale bar, 20 μm in all.

10. Assess Ca^2+^ influx and efflux of each neuron by plotting intensity over time of multiple regions of interests (ROIs) drawn on the neurites (Figure 3A).

***Optional:*** If you also use synapsin-mCherry you can draw ROIs on synapsin-mCherry positive puncta to image Ca^2+^ signals in synapses specifically.

### Live-cell imaging, DCV fusion

**Timing: 1 day**

This step visualizes single fusion events of pHluorin-labeled DCVs upon stimulation.

11. Place one coverslip containing DIV14 neurons infected with NPY-pHluorin (or BDNF-pHluorin) into an imaging chamber and add 500 μL Tyrode's solution.
12. Similar to step 7, establish perfusion, position the platinum electrodes and the glass capillary for Tyrode’s NH_4_Cl superfusion close to the neuron in focus.
13. Start a time-lapse recording with Tyrode’s NH_4_Cl superfusion at the end of the acquisition to dequench NPY-pHluorin in all DCVs (Figure 3B).

### DCV fusion events detection

**Timing: 30 min/cell**

This step provides information on semi-automatic analysis of DCV fusion events in time-lapse recordings. DCV fusion events are detected based on the standard deviation of the signal intensity throughout the stimulation period. After the automatic detection, it is possible to manually inspect the ROIs for false positives, or to manually add new ROIs.

**Figure 4:**
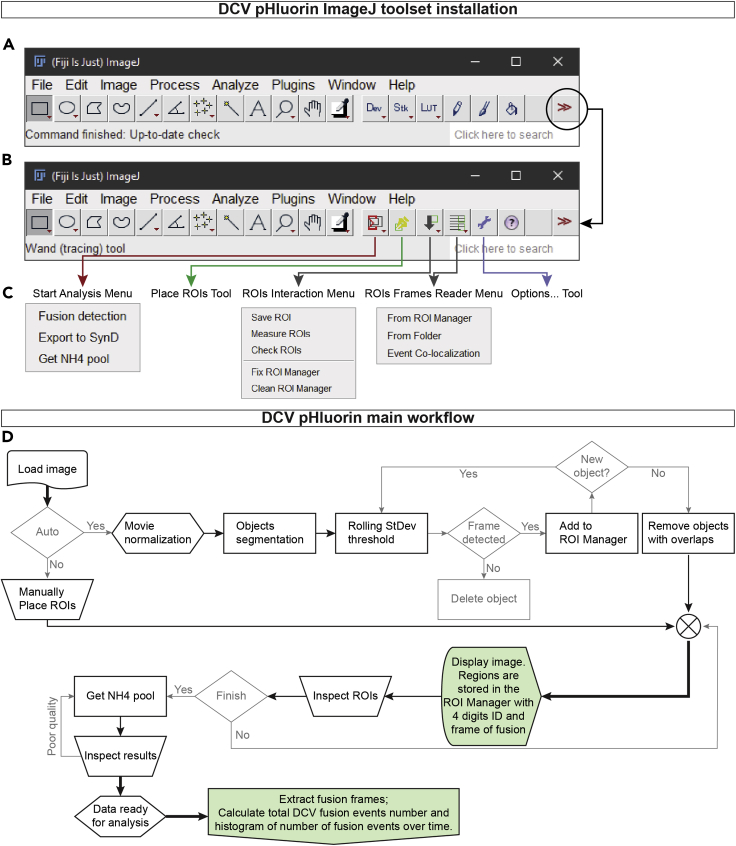
DCV_pHluorin installation and workflow (A) Fiji main interface, locate the DCV_pHluorin toolset on the toolset dropdown menu, “≫”. (B) Fiji main interface after loading the DCV_pHluorin toolset. (C) Visualization of the DCV_pHluorin toolset submenu. (D) DCV_pHluorin main workflow. Trapezoid blocks represent steps where the user is required to perform an action.

**Figure 5:**
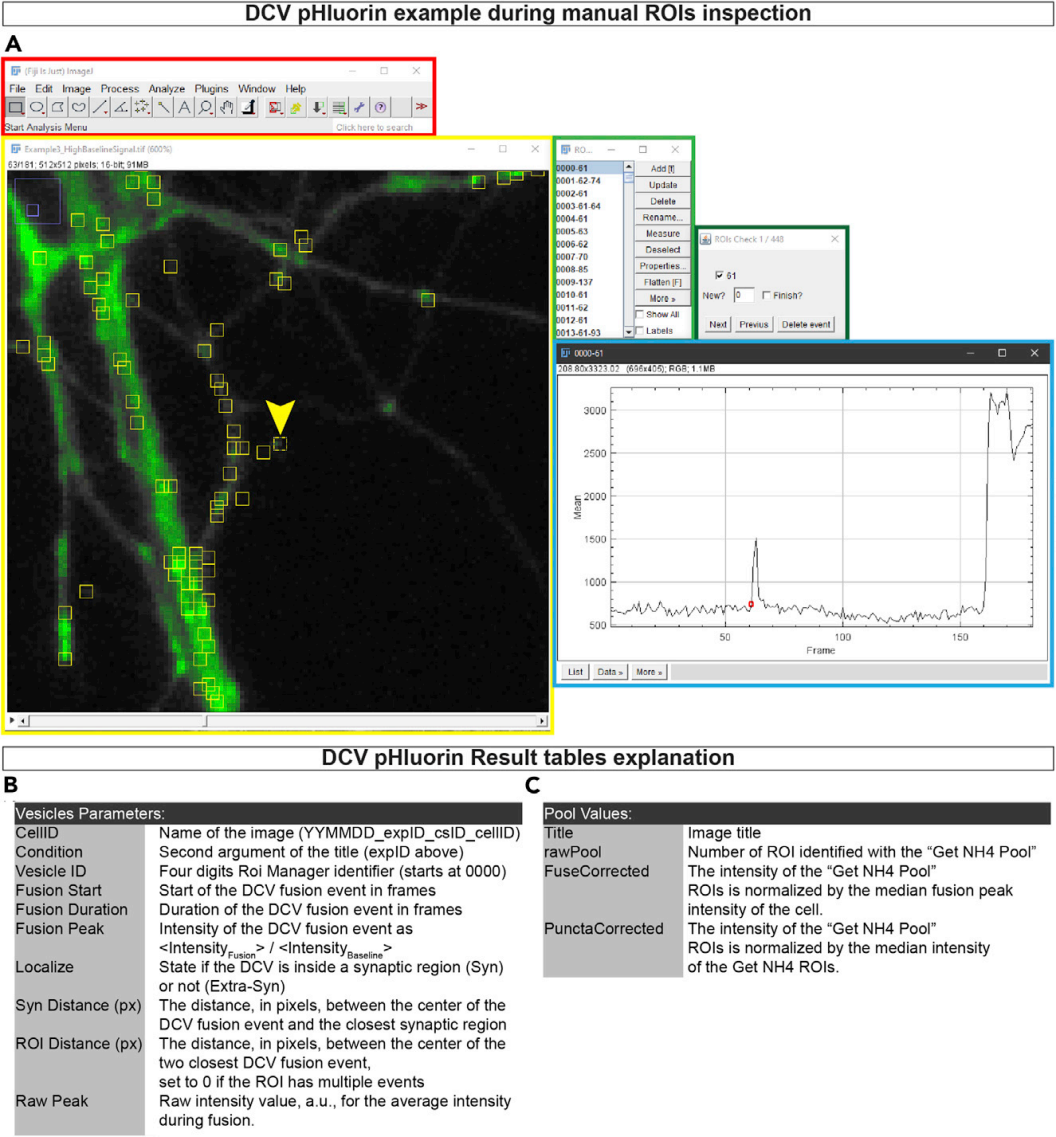
DCV_pHluorin example and result tables (A) Example Fiji interface after running the detection of DCV fusion events. In red, Fiji main interface. In yellow, the time-lapse window, the arrowhead indicates the currently selected ROI. In light-green the ROI Manager, each ROI is saved as a four digits identifier, an “-” and the fusion start frame number. In dark-green the dialog to modify the current event, from the ROIs Interaction menu > Check ROIs. In light blue the intensity over time plot of the selected ROI, the red circle indicates the start of the fusion event. (B) Explanation of the “Vesicle Parameters” result table. (C) Explanation of the “Pool Values” result table.

14. Install the DCV_pHluorin toolset by extracting the DCVpHluorin.zip file from Github in the Fiji.app/macros/toolsets folder. Start Fiji and run the toolset via selecting DCV_pHluorin in the toolset dropdown menu (Figure 4A)
  a. After the first installation, the toolset needs to be initialized by opening the “Option menu” (Figures 4B and 4C).
  b. As a final step, install the “Check ROIs” plugin via Plugin > Install Plugin… in Fiji interface. Locate the “DCVpHluorin_CheckROIs.groovy” in the Fiji.app/macros/toolsets/DCVpHluorin folder.

15. Open a time-lapse file in Fiji and manually inspect it for focus drift, bleaching, and stage movements. Open the ROI Manager and start the analysis of DCV fusion events via selecting “Fusion detection” under the “Start Analysis Menu,” and adjust the parameters as follows (Figure 3D):
  a. Movie normalization method. “B&W Opening” is set as default, use “Baseline subtraction” for noisy images.
  b. Set the “Baseline frames” and “Start of NH4 (frame)” according to your time-lapse acquisition settings. In this case 30 and 160, respectively.
  c. Select the “Number of stimulations” and the “Stimulation time (in frames)” accordingly. For more accurate measurements, it is recommended to round the duration of the stimulation period to the closest multiple of 10. For example, the 16 times 50 APs at 50 Hz last for 24 s (48 frames), so the “Stimulation time (frame)” is set to 50 frames. The program will then ask to insert the “Start of the stimulation (frame)” at a later stage.
    ***Note:*** in this case, the 16 times 50 APs at 50 Hz is considered as 1 stimulation. However, it is possible to split one stimulation in multiple blocks. Dividing the stimulation in sub-stimulations improves the detection but is more computationally intensive, thus slower and prone to memory errors.
  d. Adjust the “Smoothing radius” to improve the signal difference between background and foreground, and to reduce scatter noise.
  e. Enter the value for the “SNR” (signal to noise evaluation) as a number between 0 and 255, indicating the difference in signal between foreground and background.
    ***Note:*** it is possible to manually determine the value of the SNR by selecting “Evaluate SNR.” This will prompt the image used to detect the potential location of the DCV fusion events. Run the Fiji “Find Maxima” function with “Preview point selection” and adjust the value of the “prominence” until the majority of puncta are selected without selecting background noise. Close the “Find Maxima” and report the number on the “Evaluate SNR” dialog. The program will then restart.
  f. If the “Baseline subtraction” method was selected, insert a desired “Detection threshold” as number of standard deviations above the mean intensity signal. Only pixels with higher intensity values will be considered for ROI placement.
  g. Enter a desired “Cleaning threshold” as number of standard deviations above the mean intensity signal. This threshold is calculated as a rolling standard deviation with the value of “Baseline frames” as window size.
  h. Adjust the “ROI size” to match the observed size of the DCV fusion events.
  i. To avoid multiple detection of the same fusion event, set the “Maximum pxs overlap between ROIs” to be at most 1/3 of the total pixel area.

16. Evaluate the outcome of the detection by playing the time-lapse video. To visualize all detected regions, make sure that the “Show All” option in the ROI Manager is selected.
  a. Add any missing regions with the “Place ROIs tool;”
    ***Note:*** After the initial fusion detection, the program will name the ROI in the format of ROIID-EventStart, where ROIID is a 4 digits identifier which starts at “0000,” and the EventStart is the frame number where the signal rose above the detection threshold. For manually placed events the number corresponds to the frame selected by the user. EventStart can be repeated multiple times.
  b. Regions can be deleted at this step with “Alt + click” inside a ROI.
    ***Optional:*** The user can evaluate each ROI intensity and detection via the “Check ROIs” in the “ROIs Interaction Menu” (Figure 5A). The program will report the Intensity over Time (in frames), with the start of the DCV fusion event indicated with a red circle. Here the user can adjust the detected frame, remove, or add DCV fusion events.

17. Save the ROI Manager file via the “Save ROIs” in the “ROIs Interaction Menu.” Alternatively, the intensity of the individual ROIs can be calculated via the “Measure ROIs” function, which will report a comma separated file with the different ROIs as columns and the time-lapse frames as rows. This file can be stored for further use.
18. Once the DCV fusion events are detected and the ROI Manager file is saved, calculate the total number of pHluorin-labeled DCVs by doing the following:
  a. Make sure that the ROI Manager is open and empty, without any selections on the image;
  b. On the “Start Analysis Menu” select “Get NH4 pool.” An option dialog will appear with the same options as in step 15.
  c. The important parameters are the “Start of NH4 (frame)” and “SNR.” If the value of the SNR is set to 0, the program will calculate the SNR value based on the intensity during NH_4_Cl superfusion. Alternatively, it is possible to manually determine the SNR value selecting “Evaluate SNR” as in step 15e.
19. Evaluate the outcome of the detection by observing both the pHluorin response in the time-lapse, as well as on the “MAX” figure, which is the normalized maximum intensity projection used to detect the pHluorin positive DCVs. The program will automatically save the ROI Manager file and the list of ROI intensities in the same folder as the image file.
20. If synapsin-mCherry was used to identify synapses it is possible to create a synapse mask with the following steps:
  a. Duplicate the image and invert the gray values, in order to have a black object on white background;
  b. Perform a morphological opening by applying a minimum filter, a maximum filter, and a Gaussian blur filter of the same radius, preferably of roughly the same size as the synapses in pixels;
  c. Subtract the morphological opened image from the original and threshold the result using the “Triangle” method from Fiji “Auto threshold;”
  d. Manually inspect the synapse mask and delete any extra objects.
21. Once the detection for all recordings is completed, collect all the data via the “ROIs Frame readers Menu” and select “From Folder.” A dialog will appear asking the “baseline frames” used to calculate the baseline intensity level to estimate the DCV fusion duration, the start of the NH_4_Cl superfusion, and whether to calculate the “Synaptic location” of the fusion events, and the estimation of the total amount of pHluorin-labeled DCVs (“Pool Estimation”). This will result in a table with one entry per single DCV fusion event, and one table with three estimations of the total DCV pool (Figure 5B).

***Note:*** During data collection the program will look for the ROI Manager files of the DCV fusion events, then it will automatically calculate the DCV fusion duration and the DCV fusion intensity. If “Synaptic location” was selected, the program will open the synapse mask associated to the time-lapse and calculate the distance between the center of the DCV fusion event to the closest synaptic area. For better performance it is convenient to organize the data so that all time-lapse files and their ROI Manager files are in one single folder. Another folder will contain the NH_4_Cl response csv files and the associated ROI Manager files, and a third folder with the images of the synapse mask.

## Expected outcomes

A single hippocampus will yield between 1.5–3.0 × 10^5^ neurons/mL, so dissecting half a brain is enough for one experiment. After 2 days in culture (DIV2), neurons attached to the astrocyte microdots forming neurites can be observed (Figure 1B). At DIV9, neurons should have a visible, complex dendritic arborization populating roughly 2/3 of the astrocyte microdot. The astrocyte microdot islands should be round with smooth edges and fully populated with astrocytes. Some microdot islands will have no neurons or harbor multiple neurons. For quantitative DCV fusion analysis select the island harboring one single neuron.

During a typical Fluo-5 based live-cell imaging experiment, neurons should have a diffuse, low fluorescence signal, just above the signal from the underlying astrocytes. During stimulation, the signal should uniformly increase in the neuron, with the soma as the brightest spot, indicating Ca^2+^ influx into the cell. In response to neurotransmitter release from the neuron, it is possible to observe a wave of increase in fluorescence from the underlying astrocytes. Ca^2+^ efflux from the neurons should be fast, and visible for all of the 16 trains of APs, reaching a 70%–80% fluorescence intensity decay at the end of the stimulation.

For NPY-pHluorin based live-cell imaging experiments, the neurons will have a faint diffuse fluorescence signal that distinguishes the neurites from the underlying astrocytes. The soma will have a higher signal intensity due to the NPY-pHluorin molecules transiting through the *trans-*Golgi network, which is not acidic. Some non-acidic, pHluorin-labeled vesicles can be observed, which are trafficking along the neurites, as well as some bright puncta that could reflect pHluorin accumulation at the plasma membrane, the extracellular matrix, or in the astrocytes. During stimulation, DCV fusion events should be visible during the recordings, however depending on the intensity scaling of the recording preview, this signal might not directly be visible by eye. During Tyrode’s NH_4_Cl superfusion, the entire DCV population should be visible within 1 s from the opening of the pinch valve with vesicles moving along the neurites.

Using the 16 times 50 APs at 50 Hz stimulation paradigm, the expected number of DCV fusion events per cell is between 30 and 200, however it is possible to observe numbers up to 1,000 and below 10. The intensity of the individual fusion events, a measure of the amount of NPY-pHluorin inside the vesicle, ranges between 1.5 and 3 ΔF/F_0_ units above the baseline.

## Quantification and statistical analysis

### Data exclusion criteria

1. Neurons should respond to electrical stimulation with a sharp rise in intracellular calcium levels that should decrease rapidly after stimulation back to baseline levels. Based on our experience, if more than 30% of the tested neurons do not show Ca^2+^ influx or efflux upon electrical stimulation, it is recommended to discard the culture. Ca^2+^ is the essential trigger for DCV fusion (Hartmann et al., 2001; de Wit et al., 2009). Therefore, insufficient Ca^2+^ influx or efflux contributes to variation in DCV fusion numbers. In addition, maintaining a proper Ca^2+^ concentration gradient is essential for neuronal health (Brini et al., 2014).
2. Individual time-lapse recordings are excluded from quantification If:
  a. There are no detectable puncta after Tyrode’s NH_4_Cl superfusion. Neurons typically contain between a 1,400 and 18,000 NPY-pHluorin-labeled DCVs (Persoon et al., 2018) that appear as bright, ± 0.6 μm (3 px) puncta during Tyrode’s NH_4_Cl superfusion.
  b. There is noticeable focus drift.
  c. Cell debris, or other fluorescence objects are hovering on top of the neuron.
  d. There is a large number of non-acidic, already bright, pHluorin-labeled vesicles in the neuron.
  e. DCVs are not visibly trafficking upon Tyrode’s NH_4_Cl superfusion.
3. For correct total DCV pool estimates it is important that pixels (outside the soma) are not saturated upon NH_4_Cl superfusion. Time-lapse recordings are excluded if this is the case.

### DCV fusion events data extraction

4. Import the data generated with DCV_pHluorin into a computational software, such as MATLAB, R, or Python.
5. The “Fusion Start” value corresponds to the frame where a DCV fusion event was detected. To get the time in s of the start of the fusion event use (FusionStart−1)/F where F ≡ Imaging frequency. Then, generate a histogram of the DCV fusion events per cell.

***Note:*** Use an adequate bin size to represent the data. The ultimate goal is to avoid having too many 0 data points per bin. For 2 Hz data, a binning of 0.5 s or 1.5 s is recommended. This is because with 0.5 s the binning is per frame, while with 1.5 s the binning spans from one train of APs to the next one.

6. Calculate the cumulative count based on the histogram.
7. Extract the total number of DCV fusion events per cell and normalize it to the total DCV pool to obtain the DCV Release Fraction. DCV_pHluorin offers 3 different pool calculations, “Raw Pool,” “Fused Corrected,” and “Puncta Corrected.” It is important to carefully choose which one to use based on the overall quality of the signal during Tyrode’s NH_4_Cl superfusion. If the intensity per individual DCV fusion event is comparable to the intensity of single DCV puncta during Tyrode’s NH_4_Cl superfusion, then the “Fused Corrected” offers the best approximation of the number of pHluorin-labeled DCVs. If the intensity per individual DCV fusion event is lower than single DCV puncta during Tyrode’s NH_4_Cl superfusion, then it is suggested to use the “Puncta Corrected.”
8. Each individual neuron is considered as n = 1 for statistical evaluation. It is recommended to analyze at least 30 neurons derived from at least 3 independent cultures.
9. The different conditions are evaluated according to statistical standards. If the data is normally distributed, the difference in mean is evaluated using a Student t-test for experiments evaluating 2 conditions, or analysis of variance (ANOVA) followed by Tukey’s *post-hoc* correction for evaluating 3 or more conditions. If the data is not normally distributed the equivalent nonparametric tests, Kruskal-Wallis test and Mann Whitney U test, are used.

***Optional:*** It is possible to normalize to the total number of fusion events to observe differences in fusion kinetics between different conditions. DCV_pHluorin also generates several parameters which might be of interest, such as DCV fusion event duration, DCV fusion event location (synaptic or extra-synaptic), and the distance of the DCV fusion event from the closest synaptic location and the closest other DCV fusion location.

## Limitations

This protocol is optimized for the quantification of the number of DCV fusion events, their duration, and localization, in isolated neurons. Because of the heterogeneity of the neuronal cell types present in the culture, detecting small (<20%) effects might be challenging. Therefore, it is recommended to perform a power analysis to calculate the number of independent experiments required to obtain sufficient power. In our experience, a significant difference of 30% or more requires 30 neurons from 3 independent cultures. Selection of a specific neuronal cell type by expressing DCV fusion reporters under a specific promoter, such as somatostatin, parvalbumin (for GABAergic neurons), or CaMKII (for mature glutamatergic neurons) may reduce variability.

## Troubleshooting

### Problem 1

Neurons do not respond to electrical stimulation (step 8 in Step-by-step method details).

### Potential solution

First, make sure that the electrodes are ∼ 1 mm from the surface of the coverslip and that they are surrounding the neuron in the field of view. If neurons are still not responding to electrical stimulation, calibrate the stimulus strength required to depolarize the membrane. To do so, label neurons with the calcium indicator Fluo5-AM and stimulate with 1 ms pulses starting at 100 mA, and decrease the current until the minimum current is reached that reliably elicits Ca^2+^ influx during 1 ms pulses.

### Problem 2

No DCV fusion upon stimulation (step 13 in Step-by-step method details).

### Potential solution

DCV fusion is variable between neurons. Therefore image at least 10 neurons before concluding that there is no DCV fusion upon stimulation. It is also important to exclude low expression of the reporter, by carefully inspecting the response to Tyrode’s NH_4_Cl application. The response should be fast, reaching a maximum intensity within 1 s after the application, and the entire neurite arbor should contain labeled vesicles. It is also important to notice that high expression levels of the reporter could cause more DCVs to be less acidic. This will also affect the number of DCV fusion events upon stimulation. Therefore, it is suggested to titer the number of lentivirus particles to add to the culture as the minimum amount that reliably infects ∼90% of the neurons on a coverslip.

### Problem 3

DCV_pHluorin detects the same DCV fusion event in multiple ROIs (step 16 in Step-by-step method details).

### Potential solution

It is possible to observe multiple DCV fusion events less than 1 px apart in regions where the synapse density is higher, or with multiple neurite crossings. To avoid detection of the same DCV fusion event in multiple ROIs, it is recommended to set the “Maximum pxs overlap between ROIs” to be at most 1/3 of the total area of the ROIs. In case of 3 × 3 px ROIs, the “Maximum pxs overlap between ROIs” would then be of 3 pxs. By doing so, even if one DCV fusion event might span multiple ROIs, only the ROIs that contains all the pixels associated with the DCV fusion event will have a mean intensity value high enough to be detected. It is recommended to manually inspect potential overlap of ROIs via the “Check ROIs” function, especially in fusion dense areas.

### Problem 4

Non-homogeneous fluorescence signal intensity in different regions of the neurite arbor (steps 10, 13, and 15 in Step-by-step method details).

### Potential solution

Different signal intensities across different regions of the neurite arbor could be caused by a miss-aligned illumination path. To test if this is the case, it is recommended to visualize a uniformly distributed protein, such as cytosolic GFP. The problem should be solved by aligning the light path.

If the uneven illumination, or signal intensity, leads to overrepresentation of the higher signal areas, it is recommended to adjust the “Movie normalization” settings. By using the “Baseline subtraction” Fiji will calculate the average intensity value of the first X frames (X set by user in “Baseline Frames” option). It will subtract this average intensity value from all frames. This method works well for noisy images, but it is sensitive to uneven signal intensities, thus favoring regions with high signal.

An additional option for "movie normalization" is the "B&W opening" option. This uses the information of the pixels surrounding the pixels of interest by a value “Smoothing radius” to first perform a maximum filter, then a minimum filter, and lastly a Gaussian Blur filter. Since the “B&W Opening” scales pixels based on local information compared to the entire image, it is more suitable for images with uneven illumination or signal intensity. In addition to both normalization methods, the detection is done on the standard deviation of the signal over time. Using the standard deviation ensures that high intensity regions are not overrepresented compared to low intensity regions.

### Problem 5

Non-acidic structures are visible before NH_4_Cl perfusion (step 13 in Step-by-step method details).

### Potential solution

As stated in problem 2, too high expression levels of the DCV reporter construct could cause DCVs to be less acidic. In addition, when designing new DCV reporter constructs by tagging other neuropeptides with pHluorin it is possible to observe mistargeting to other organelles. For example, mistargeting to reticular or tubular structures has been observed for NPY tagged with EGFP (Nagai et al., 2002). To test if your DCV reporter of interest targets to DCVs it important to first validate the sequence of your construct. Next, it is recommended to verify efficient targeting to DCVs by co-immunolabeling with endogenous DCV markers (Persoon et al., 2018). If your reporter construct is indeed mistargeting it is recommended to try and reduce the construct length to a minimal functional construct (for example only containing the signal peptide and N-terminal domain of the pre-propeptide) and subsequently validate the correct targeting.

## Resource availability

### Lead contact

Further information and requests for resources and reagents should be directed to and will be fulfilled by the Lead Contact, Ruud F. Toonen (ruud.toonen@cncr.vu.nl).

### Materials availability

This study did not generate new unique reagents. Plasmids generated in this study are available upon request.

### Data and code availability

The DCV_pHluorin code is available in GitHub at https://github.com/alemoro
